# Clinical and seasonal pattern of dengue: Persistent hyper-endemicity of a vector borne disease from Southern-West Coastal India

**DOI:** 10.12688/f1000research.126845.1

**Published:** 2023-07-11

**Authors:** Darshan BB, Ramesh Holla, Bhaskaran Unnikrishnan, Basavaprabhu Achappa, Robin Poovattil, Ashir Sharma, Shawna Simmy, Suryansh Prateek

**Affiliations:** 1 Manipal Center for Infectious Diseases, Prasanna School of Public Health, Manipal Academy of Higher Education, Manipal, Karnataka, India; 2 Kasturba Medical College, Mangalore, Manipal Academy of Higher Education, Manipal, Karnataka, India

**Keywords:** Dengue, Record based Study, Tertiary care Hospital, South India

## Abstract

**Background:** Dengue is an emerging global viral disease with an increase 30-fold in incidence in the past fifty years. In the past decade it was restricted to only few a states of South and Northern India but in the recent past it has affected almost all the states in India. The objective of this study was to assess the clinical profile, trends and outcome of dengue cases.

**Methods:** This retrospective record based cross-sectional study was conducted in tertiary hospitals, Mangaluru in Southern India. The study population included all clinical dengue positive cases over a period of five years. Information from pre-recorded case sheets were used for data collection. The data collected was entered and analyzed in SPSS Version 20. Results were expressed in percentages, means and graphs.

**Results:** The study included 401 dengue cases. Most cases were in the age range of 20-40 years with a male to female ratio of 3:2. Overall seropositivity rate was 23.94% with High IgM prevalence. Monthly distribution showed a maximum incidence in the months of June and July and minimum incidence in January and February. Among the study participants, 91.5% of patients recovered completely and 1.7% of patients had died. 8.7% of patients were discharged against medical advice.

**Conclusions:** Dengue continues to be major public health problem which indirectly hints towards the hyper endemic nature of this disease in this part of the globe affecting mainly the working age group. Low seropositivity with High IgM prevelance makes dengue an important differential for febrile illness of vague nature and invokes the need for robust public health response to curb the hyper-endemicity.

## Introduction

Dengue is an emerging global viral disease with an increase in incidence of 30 times in the past fifty years. Nine countries were affected before the 1970s but now more than 100 counties have severely been affected with dengue epidemics with the Southeast Asian region and the Western Pacific region being the most severely affected. 390 million cases of dengue occur annually with 96 million showing clinical manifestation. ^1^

The first case of dengue was reported by Benjamin Rush in 1789. Up until the middle of 20 ^th^ century it was restricted to few geographical locations, but with the population movement during the Second World War there were recurrent epidemics with the re-emergence of the disease. ^2^

Even though the first epidemic of dengue was reported from Chennai in India, the virologically proven epidemic occurred in Kolkata in the 1950s. There were cases of dengue hemorrhagic fever and dengue shock syndrome in Delhi and Lucknow in the year 1996, thereafter with a cyclical pattern occurring every 2-3 years. ^2^

In the past 20-30 years it was mostly restricted to only a few states of South and Northern India but in the last decade it has affected almost all the states of India. ^3^

Dengue is viral vector borne disease spread by the day-biting endophilic Aedes mosquito. Its clinical features tend to be vague and nonspecific, ranging from fever to hemorrhage to shock with no specific treatment but supportive care. ^4^^,^^5^

Geographically, dengue is more prevalent in tropical countries like India. With that background understanding, we conducted our study in the coastal part of South India where developmental activities are on a rise with rapid urbanization where the study population is at a high risk of being affected with dengue. Our study was conducted to determine the socio-demographic and clinical profile along with the disease outcome of dengue patients. As information regarding the trend, burden and distribution of the disease is vital to plan disease control strategies and optimum utilization of the resources, our study aims to further contribute to the knowledge base regarding this disease.

## Methods

### Study design

Retrospective record-based Cross sectional study design

### Study location

The study was conducted at Government Wenlock Hospital, a tertiary care teaching hospital affiliated to Kasturba Medical College, Mangalore.

Being a hospital which receives a confluence of patients from neighboring districts of Karnataka and from northern parts of Kerala, our study population well represents the burden of dengue in the South Western part of India.

### Study population

The study population was dengue patients admitted to Government Wenlock Hospital within the time frame of five years from 2013 to 2017.

**Inclusion criteria**: Clinically confirmed Dengue cases admitted at the above-mentioned time period.

**Exclusion criteria**: All case sheets with inadequate or incomplete data were excluded from the study.

### Ethics and consent

The IEC (Institutional Ethics Committee) of Kasturba Medical College, Mangalore (Manipal Academy of Higher Education) has reviewed the study and has granted approval prior to the onset of the study. Owing to the nature of study design as retrospective record based study, informed consent was waived by the Ethics Committee. Confidentiality of the present study data was maintained in accordance with the Declaration of Helsinki.

### Data collection

Case sheets of Dengue positive patients fitting the inclusion criteria were carefully analyzed. Relevant demographical, clinical and biochemical parameters were recorded onto the data collection sheet.

### Analysis

Data collected was then analyzed with SPSS version 20. Results have been expressed in means, proportions and standard deviations.

## Results

### Demographic profile

Our study was able to reinforce the prevalence of certain demographic trends that has been observed among patients affected with Dengue. Out of the 401 patients studied, 245 (61%) were males with a male to female ratio of 3:2. Majority of the cases fell under the age bracket of 20-40 years (169 cases, 42.1%) ( Table 1). Certain demographic details such as occupation, marital status, place of residence were not available for all the patients and hence were not reported upon in our results.

**Table 1.** Baseline characteristics of dengue patients (n=401).

Baseline characteristics	Number	Percentage
**Age group (years)**		
< 20	116	28.9
20-40	169	42.1
40-60	097	24.1
>60	019	04.9
**Gender**		
Male	245	61.0
Female	156	39.0
**Duration of stay (days)**		
<5	247	61.6
6-10	113	28.2
>10	041	10.2

### Clinical and laboratory findings

The most common and consistent clinical feature with which most of the patients presented with was fever (398, 99.3%) proceeded by chills and rigor (256, 63.8%) followed by myalgia (194, 48.4%) ( Table 2).

**Table 2.** Distribution pattern of the chief presenting complaints (n=401).

Clinical presentation	Number *	Percentage
Fever	398	99.3
Chills and Rigor	256	63.8
Myalgia	194	48.4
Headache	171	44.6
Vomiting	152	37.9
Pain abdomen	090	22.4
Arthralgia	059	14.7
Diarrhoea	031	07.7
Melena	018	04.5
Breathlessness	013	03.2
Rash	012	02.9
Hematemesis	012	02.9
Haematuria	007	01.7

* Multiple responses.

In line with expected trends, it was observed that most of our cases (n=368, 95.8%) had thrombocytopenia. 81.6% of patients had elevated SGOT and 48% had elevated SGPT enzyme levels indicating certain degree of hepatocyte injury during the acute phase of the infection.

However, a closer look at the seropositivity rate amongst the study population posed some interesting queries. Out of the 401 cases, only 96 cases showed elevated IgG or IgM (total seropositivity rate of 23.94%). Separately, seropositivity rate of IgM was 22.9% and of IgG was 2.7%. Sex wise distribution shows 25.7% seropositivity in males and 22.72% seropositivity among females.

But amongst the seropositive patients 57.29% (55 cases) were female and 42.7% (41 cases) were male ( Table 3).

**Table 3.** Laboratory profile of dengue patients.

Lab parameter (n=384)	Number	Percentage
Platelet Count (< 150000/micro litre)	368	95.8
SGOT (>40 IU/L)	298	81.6
SGPT( >56 IU/L)	180	48.0
MCHC(<33 g/dl)	204	91.4
**Seropositivity (n=401)**		
Overall seropositivity (IgM or IgG)	96	23.94
IgM seropositivity	92	22.90
IgG seropositivity	11	02.70

Frequency distribution of seropositive cases with respect to age showed that most cases (36, 37.5%) were in the age group of 20-40yrs followed by <20yrs (32, 33.3%).

### Clinical outcome

In our study, it was observed that despite the state of presentation and clinical course the mass preponderance was towards recovery with a recovery rate of 91.5% (n=367). Case fatality rate was recorded to be 1.7% ( Table 4).

**Table 4.** Clinical outcome of dengue patients (n=401).

Outcome	Number of cases	Percentage
Recovery	367	91.5
Death	007	01.7
Discharge Against Medical Advice (DAMA)	027	06.8

### Seasonality

**Figure 1.**
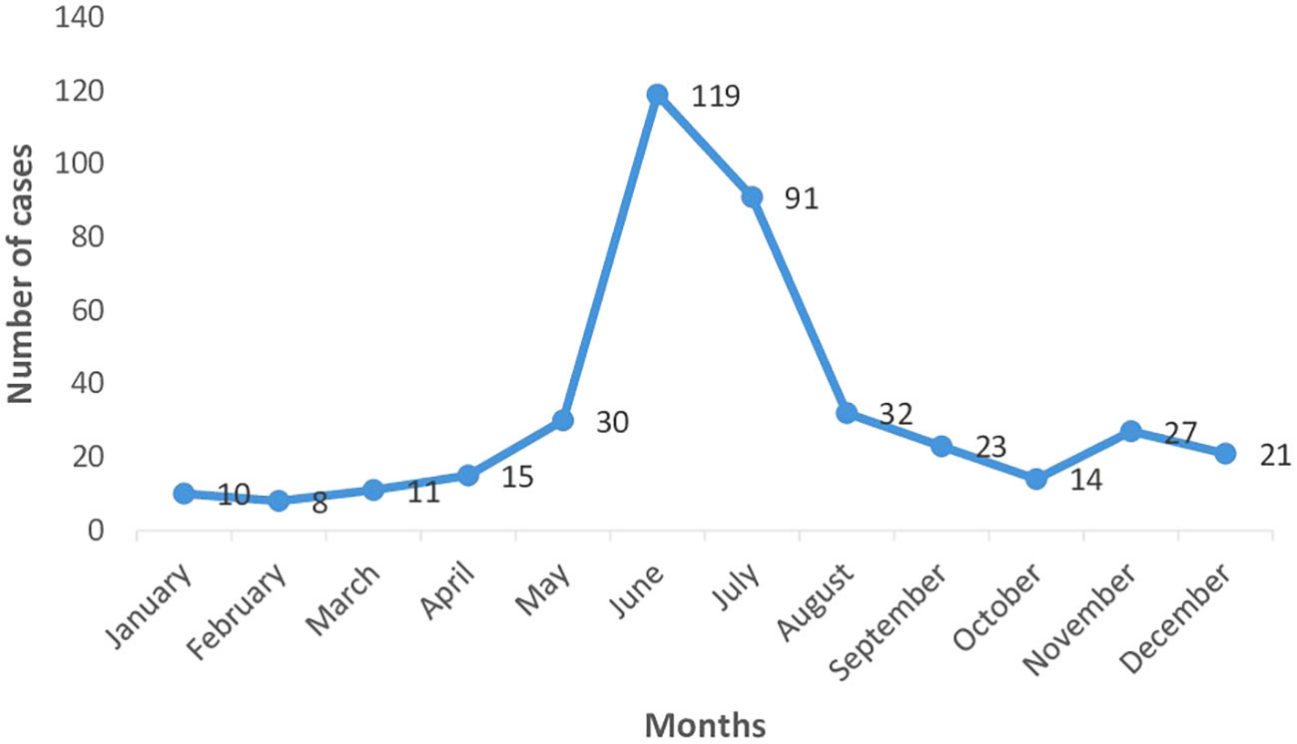
Month wise distribution of dengue cases (n=401).

Dengue being a vector borne disease was expected to boom in accordance with the monsoon seasons and our study was able to confirm this. As depicted by Figure 1 [Month wise distribution of dengue cases (n=401)], most of the cases were found to have been admitted during the months of June and July with a declining trend both pre- and post-monsoons. The months of January to April were shown to have a consistently low prevalence.

## Discussion

Dengue is an upcoming and swiftly spreading vector borne disease that has taken strong hold in India. ^4^ Owing to the complicated interplay between the host, agent, vector, and environmental conditions, the number of cases in India has consistently increased substantially over the past ten years. ^6^ It is now even considered as a hyper endemic disease in certain parts of India. As Das *et al.* demonstrated in their study, the available data on dengue prevalence in India is just the tip of the iceberg and that further weighs down the preventive measures taken to reduce the brunt of the burden. ^7^ Having recognized the need for comprehensive stratified data on dengue prevalence and its trends to further the knowledge database in Indian population, we focused our study to assess the various clinical, sociodemographic and climactic factors.

Our study was able to demonstrate a significant male preponderance (male = 61%, females = 39%) in incidence with a male to female sex ratio of 1.5:1. Similar observations have been made by a multitude of studies, yet a conclusive reasoning has not yet been attributed to this trend. ^8^ Doke and Pawar has attributed this finding to the nature of dressing among women which reduces the amount of skin exposure. ^9^ Other studies have explained this as a product of reporting bias among females as it has been seen that traditional practitioners are the first point of contact for the large majority of symptomatic female population. ^10^ A few studies even postulated that this disparity could be due to the skewed sex ratio leaning towards the side of males in the general population. ^11^

Apart from the male preponderance, our research found that the productive working class aged 20-40 years was the age group most affected. Owing to the declining rate of incidence with advancing age, it could be said that infants, adolescents and young adults are at higher risk of developing dengue. Similar trend has been observed in other studies wherein they found that dengue is a disease that primarily affects the children and the young adults. ^3^^,^^12^^–^^15^

Combined, the age and sex distribution findings could be attributed to the complex interplay between outdoor nature of work (among men), ^9^ dressing patterns (among women), skewed sex ratio and diurnal feeding habbits of Aedes aegypti. ^4^

Most of the cases were found to have occurred in the months of June and July, with the maximum being 120 cases. A study conducted by Kumar *et al.* in a coastal city of Karnataka revealed the maximum cases were found to have occurred in the month of September while a meta-analysis conducted by Ganeshkumar *et al.* found that most cases were seen in the monsoon and post monsoon seasons. ^13^^,^^16^ The peri-monsoon seasonality of dengue has also been promptly emphasized by a number of studies which implicates that there is a strong correlation that exists between temperature and humidity to the favourable breeding conditions for the mosquitoes. ^3^^,^^13^^,^^14^ During rainy season environmental changes such as artificial water stagnation, especially in low lying areas, labour settlements and small collection of water in tyres and flowerpots act as favourable breeding grounds for the vector which conclusively explains the precipitous climb of dengue cases during June to September. ^14^

The clinical picture of patients in our study revealed that fever was present in almost all cases (99.3%) followed by chills and rigor (63.8%). Haematological symptoms included hematemesis and haematuria which were present in 12% and 7% of cases respectively. A meta-analysis conducted on a global scale on dengue outbreaks came to an almost similar picture with fever (98.1), chills (65.3), myalgia (64.2), arthralgia (53.6), body pain (67.2), vomiting (39.8) etc. with similar haematological symptoms like haematuria and hematemesis which were seen in 5% and 13.4% of cases respectively. ^17^

Liver functions test showed abnormal rise in almost 99% of patients of SGPT and SGOT levels but SGOT levels were more prominent in most patients as compared to SGPT, similar research done in Punjab in 2007 had similar results with 98.9% of patients showing a rise in either of SGPT or SGOT levels indicating a strong heptic predeliction for dengue virus. ^18^ A dedicated study regarding dengue and hepatopathy also revealed similar results of elevated SGOT and SGPT with the former being more elevated than the latter. ^19^

Thrombocytopenia is the most common laboratory finding in dengue patients and is referred to as an early marker and prognostic factor for the management and recovery of dengue fever. ^20^ Of 384 dengue patients studied, 368 patients had thrombocytopenia (platelet count less than 100,000 per mm ^3^).

Our study revealed a seropositivity rate of 23.94% which is similar to another study conducted by Kalita *et al.* where they repoted a seropositivity of 14.85%. ^21^ Along with having a low seropositivity rate, it was seen that High IgM prevelance was noted in our study. As concluded by Eshetu *et al.*, high IgM prevelance is indicative of active transmission of dengue which could account for the hyperendemic status of dengue in Southern India. ^22^

Our study included 401 serologically confirmed dengue cases out of which 367 cases (91.5%) were found to have recovered completely. Seven deaths were recorded (1.7%), and 27 cases (6.7%) were found to have been discharged against medical advice. A meta-analysis conducted in India, found that the pooled CFRs of the studies was 2.6% which was in line with findings of the present study. ^16^

## Conclusion

Dengue remains to be a major public health problem which indirectly hints towards the hyper endemic nature of this disease in this part of the globe, affecting mainly the working age group. With vague nonspecific clinical features combined with low seropositivity rate, it is of paramount importance to keep dengue as a key differential when a patient presents with febrile illness of nonspecific nature. Clinical judgement with classical biochemical parameters involving platelets as demonstrated and reinforced by our study should take priority over serological status of the patient when it comes to making a diagnosis of dengue or excluding it. High IgM seropositivity also hints at the fact that a strong and robust vector control programme must be implemented to at least make a dent in the hyperendemic status of dengue especially during the monsoon seasons.

## Data Availability

Figshare: Data.xlsx (demographic and medical information of patients), https://doi.org/10.6084/m9.figshare.21257040.v1. ^23^ Data are available under the terms of the Creative Commons Zero “No rights reserved” data waiver (CC0 1.0 Public domain dedication).
