# Supplementary material for: Clinical and seasonal pattern of dengue in a tertiary care hospital of South West India
Source: F1000Res. 2024 Mar 11;12:817. Originally published 2023 Jul 11. [Version 3] doi: 10.12688/f1000research.126845.3 (PMC11016885; doi:10.12688/f1000research.126845.3)
Supplement: Peer review history

# Reviewer report: Reviewer response for version 3

**DOI:** 10.5256/f1000research.163357.r254559

**Published:** 2024-04-14

**Authors:** Biju Soman

**Affiliations:** 1 Achutha Menon Centre for Health Science Studies, Sree Chitra Tirunal Institute for Medical Sciences and Technology, Thiruvananthapuram, Kerala, India

This analysis has the limitations of being a hospital-based study located in a bordering district of Karnataka.  Its catchment area is unclear and could often be affected by interstate travel restrictions. However, this work of budding researchers could point to some overarching trends of dengue in the region.

Is the work clearly and accurately presented and does it cite the current literature?

Partly

If applicable, is the statistical analysis and its interpretation appropriate?

Not applicable

Are all the source data underlying the results available to ensure full reproducibility?

Partly

Is the study design appropriate and is the work technically sound?

No

Are the conclusions drawn adequately supported by the results?

No

Are sufficient details of methods and analysis provided to allow replication by others?

Partly

Reviewer Expertise:

Epidemiology, Infectious Disease Modeling, GIS in public Health, Data Science, Health Technology Assessment

I confirm that I have read this submission and believe that I have an appropriate level of expertise to confirm that it is of an acceptable scientific standard.

# Reviewer report: Reviewer response for version 2

**DOI:** 10.5256/f1000research.162844.r249704

**Published:** 2024-02-29

**Authors:** Biju Soman

**Affiliations:** 1 Achutha Menon Centre for Health Science Studies, Sree Chitra Tirunal Institute for Medical Sciences and Technology, Thiruvananthapuram, Kerala, India

Thank you for the earnest attempt to comply with the reviewers' suggestion. I feel that a bit more tidying up would be helpful.  The manuscript still needs language correction. A couple more ways to improve the manuscript include the following. 

- Include the study details in the methods section, like how the cases are enrolled for the study, the criteria used for diagnosis of dengue, etc.  Then only the mention of seropositivity in our results session or in the abstract makes any sense. 
- Please be careful in using epidemiological terms like 'incidence.'  This study, being a hospital-based retrospective analysis, cannot comment on the incidence of dengue. (eg: "Monthly distribution showed a maximum incidence in the months of June and July and minimum incidence in January and February.")

Is the work clearly and accurately presented and does it cite the current literature?

Partly

If applicable, is the statistical analysis and its interpretation appropriate?

Not applicable

Are all the source data underlying the results available to ensure full reproducibility?

Partly

Is the study design appropriate and is the work technically sound?

No

Are the conclusions drawn adequately supported by the results?

No

Are sufficient details of methods and analysis provided to allow replication by others?

Partly

Reviewer Expertise:

Epidemiology, Infectious Disease Modeling, GIS in public Health, Data Science, Health Technology Assessment

I confirm that I have read this submission and believe that I have an appropriate level of expertise to confirm that it is of an acceptable scientific standard, however I have significant reservations, as outlined above.

# Response

**Published:** 2024-03-03

**Authors:** Ramesh Holla

**Affiliations:** Kasturba Medical College, Mangalore, Manipal Academy of Higher education, Manipal, India, India

Point to point response to Reviewer's comments:  

**Dr. Biju Soman**

 1. **Comment: **Include the study details in the methods section, like how the cases are enrolled for the study, the criteria used for diagnosis of dengue, etc.  Then only the mention of seropositivity in our results session or in the abstract makes any sense.

**Author’s Response: **We agree with your comment. As per the suggestion, Necessary changes have been done in the methodology section.

**Action Taken in the manuscript: **As per the suggestion, necessary changes have been done in the methodology section.

- All dengue patients admitted to Government Wenlock Hospital within the time frame of five years from 2013 to 2017 were included in the present study.
- IgM Capture ELISA and Dengue Non-structural Protein NS1 antigen test which is a rapid test kit, were used for the diagnosis of Dengue in the study participants.

2. **Comment: **Please be careful in using epidemiological terms like 'incidence.'  This study, being a hospital-based retrospective analysis, cannot comment on the incidence of dengue. (eg: "Monthly distribution showed a maximum incidence in the months of June and July and minimum incidence in January and February.")

**Author’s Response: **We are in complete agreement with your observation. Necessary changes have been done in the result section of the manuscript. Incidence word was replaced with proportion of cases and case load in the hospital.

**Action Taken in the manuscript: **Necessary changes have been done in result section.

 3. **Comment:** The manuscript still needs language correction.****

**Author’s Response: **Manuscript was proof read and grammatical corrections were done.

**Action Taken in the manuscript: **Manuscript was proof read and grammatical corrections were done throughout the manuscript.

# Reviewer report: Reviewer response for version 2

**DOI:** 10.5256/f1000research.162844.r249703

**Published:** 2024-02-26

**Authors:** Zinia Nujum

**Affiliations:** 1 Department of Community Medicine, Government Medical College, Kollam, Kerala, India

No new comments on the revised version.

Is the work clearly and accurately presented and does it cite the current literature?

Partly

If applicable, is the statistical analysis and its interpretation appropriate?

Partly

Are all the source data underlying the results available to ensure full reproducibility?

No

Is the study design appropriate and is the work technically sound?

Yes

Are the conclusions drawn adequately supported by the results?

Partly

Are sufficient details of methods and analysis provided to allow replication by others?

Yes

Reviewer Expertise:

Neglected Tropical Diseases

I confirm that I have read this submission and believe that I have an appropriate level of expertise to confirm that it is of an acceptable scientific standard.

# Reviewer report: Reviewer response for version 1

**DOI:** 10.5256/f1000research.139295.r187600

**Published:** 2023-10-25

**Authors:** Biju Soman

**Affiliations:** 1 Achutha Menon Centre for Health Science Studies, Sree Chitra Tirunal Institute for Medical Sciences and Technology, Thiruvananthapuram, Kerala, India

This is a good attempt at looking at the data from a prominent tertiary care hospital in Mangalore. However, the inference drawn by the authors conflicts with the hospital-based nature of the data. The manuscript should be rewritten as a hospital-based study, and the authors should refrain from giving statements on hyperendemicity, etc. The following detailed comments should be kept in mind while revising the paper.

- “ *The study population included all clinical dengue positive cases over a period of five years.*” (p. 1)
  Give the exact period and the jurisdiction. If it is the Mangalore town, define it; if it is the district, specify it.
- “ *The data collected was entered and analyzed in SPSS Version 20*” (p. 1)
  Please copy edit
- “ *Results were expressed in percentages, means and graphs.*” (p. 1)
  Please copy edit
- “ *Among the study participants, 91.5% of patients recovered completely, and 1.7% of patients had died. 8.7% of patients were discharged against medical advice.*” ( p. 1)
   Better round the decimal figures to 100%
- “ *Being a hospital which receives a confluence of patients from neighbouring districts of Karnataka and northern parts of Kerala, our study population well represents the burden of dengue in the South Western part of India.*” (p. 3)
  This assumption cannot be justified. Try to present this as a hospital study; at the most, you can claim this hospital as a sentinel hospital and suggest that the trend in this hospital could reflect what is happening in and around this location. However, please remember that Wenloc Hospital can no longer claim to cater to all or even a good majority of Dengue cases in the locality. There are many other government and private hospitals in the area. Also, it would help if you did a trend analysis to state the increase, decrease or other status of Malaria cases coming to the hospital. The Yearwise distribution should be used.
- “ *In our study, it was observed that despite the state of presentation and clinical course, the mass preponderance was towards recovery with a recovery rate of 91.5% (n=367).*” ( p. 5)
  This sentence is also unclear; the manuscript will benefit from thorough copy editing.

Is the work clearly and accurately presented and does it cite the current literature?

Partly

If applicable, is the statistical analysis and its interpretation appropriate?

Not applicable

Are all the source data underlying the results available to ensure full reproducibility?

Partly

Is the study design appropriate and is the work technically sound?

No

Are the conclusions drawn adequately supported by the results?

No

Are sufficient details of methods and analysis provided to allow replication by others?

Partly

Reviewer Expertise:

Epidemiology, Infectious Disease Modeling, GIS in public Health, Data Science, Health Technology Assessment

I confirm that I have read this submission and believe that I have an appropriate level of expertise to state that I do not consider it to be of an acceptable scientific standard, for reasons outlined above.

# Response

**Published:** 2024-02-17

**Authors:** Ramesh Holla

**Affiliations:** Kasturba Medical College, Mangalore, Manipal Academy of Higher education, Manipal, India, India

Point to point response to Reviewer's comments:  

**Dr. Biju Soman**

 1. **Comment: **Give the exact period and the jurisdiction. If it is the Mangalore town, define it; if it is the district, specify it.

**Author’s Response: **We are in complete agreement with your observation. Our subset of patients are mainly residents of Dakshina Kannada district of Karnataka, Northern Kerala districts such as Kasargod, Kannur and Wayanad with a minority group of patients from other districts of Karnataka as well.

**Action Taken in the manuscript: **Necessary changes have been done in the Materials and Methods section of the manuscript.

 2. **Comment: **Better round the decimal figures to 100%.

**Author’s Response: **We concur that it is better to round the decimal figures as pointed out.

**Action Taken in the manuscript: **Figures have been rounded off to the nearest whole number.

 3. **Comment:  **This assumption cannot be justified. Try to present this as a hospital study; at the most, you can claim this hospital as a sentinel hospital and suggest that the trend in this hospital could reflect what is happening in and around this location. However, please remember that Wenlock Hospital can no longer claim to cater to all or even a good majority of Dengue cases in the locality. There are many other government and private hospitals in the area. Also, it would help if you did a trend analysis to state the increase, decrease or other status of Malaria cases coming to the hospital. The Year wise distribution should be used.

**Author’s Response: **We agree that it would be better to represent our data as a hospital-based study that reflects the Dengue burden in the coastal districts of Kerala and Karnataka. We will also take into consideration the note to add year wise distribution of Dengue cases.

**Action Taken in the manuscript: **Study title has been changed as per the suggestion **. **The results obtained have been rephrased to be a representation of a hospital-based survey. Year wise distribution of Dengue case load has been added as a graph.

 4. **Comment: **This sentence is also unclear; the manuscript will benefit from thorough copy editing.

**Author’s Response: **We agree that a thorough copy editing would help communicate the conclusions better to the reader.

**Action Taken in the manuscript: **Necessary grammatical changes have been made

 5. **Comment: **“The data collected was entered and analyzed in SPSS Version 20” (p. 1)

 Please copy edit

**Author’s Response: **We agree that this statement could be rephrased better.

**Action Taken in the manuscript: **This sentence has been rewritten

 6. **Comment: **“Results were expressed in percentages, means and graphs.” (p. 1)

 Please copy edit

**Author’s Response: **We agree that this statement could be rephrased better.

**Action Taken in the manuscript: **This sentence has been rewritten

# Reviewer report: Reviewer response for version 1

**DOI:** 10.5256/f1000research.139295.r187595

**Published:** 2023-10-25

**Authors:** Zinia Nujum

**Affiliations:** 1 Department of Community Medicine, Government Medical College, Kollam, Kerala, India

**General Comment**

 The study is a single centre study done on a topic of public health relevance, but the information presented is not new. In order to bring in an analytic perspective, I would suggest classification of dengue cases into severe and not severe based on the need for hospitalization or shock and look at the determinants of severity. Since the number of deaths is small, they may also be included as severe dengue.

**Specific comments**

 Title: The part "persistent hyperendemicity" may be taken off from the title. This study has not looked into the serotypes. It is better to keep the title simple as 'Clinical and Seasonal Pattern of Dengue in a tertiary care setting in South West India'.

 Discussion: "Our study was able to demonstrate a significant male preponderance (male = 61%, females = 39%) in incidence with a male to female sex ratio of 1.5:1" - you cannot conclude on incidence from this study, you may say that there is a male preponderance in cases of dengue reporting to this hospital.

 Results:

- Overall IgM/IgG sero-positivity is only 23%. Then how was the diagnosis confirmed in rest of the cases?
- What proportion of cases were dengue, severe dengue (DHF, DSS, Dengue with organ involvement - expanded Dengue syndrome)?

Conclusions: The conclusions are vague and tend to be written as general statements than from the results of the study **.**

Is the work clearly and accurately presented and does it cite the current literature?

Partly

If applicable, is the statistical analysis and its interpretation appropriate?

Partly

Are all the source data underlying the results available to ensure full reproducibility?

No

Is the study design appropriate and is the work technically sound?

Yes

Are the conclusions drawn adequately supported by the results?

Partly

Are sufficient details of methods and analysis provided to allow replication by others?

Yes

Reviewer Expertise:

Neglected Tropical Diseases

I confirm that I have read this submission and believe that I have an appropriate level of expertise to confirm that it is of an acceptable scientific standard, however I have significant reservations, as outlined above.

# Response

**Published:** 2024-02-18

**Authors:** Ramesh Holla

**Affiliations:** Kasturba Medical College, Mangalore, Manipal Academy of Higher education, Manipal, India, India

Point to point response to Reviewer's comments:  

**Dr. Zinia Nujum**

 1. **Comment: **Title: The part "persistent hyperendemicity" may be taken off from the title. This study has not looked into the serotypes. It is better to keep the title simple as 'Clinical and Seasonal Pattern of Dengue in a tertiary care setting in South West India'.

**Author’s Response: **We agree with your comment. As per the suggestion title has been modified.

**Action Taken in the manuscript: **As per the suggestion title has been modified.

 2. **Comment: **Discussion: "Our study was able to demonstrate a significant male preponderance (male = 61%, females = 39%) in incidence with a male to female sex ratio of 1.5:1" - you cannot conclude on incidence from this study, you may say that there is a male preponderance in cases of dengue reporting to this hospital.

**Author’s Response: **We value your suggestion. Necessary changes have been done in results and discussion section.

**Action Taken in the manuscript: **Necessary changes have been done in results and discussion section.

 3. **Comment: **Overall IgM/IgG sero-positivity is only 23%. Then how was the diagnosis confirmed in rest of the cases?

**Author’s Response:  **As a first line of diagnosis, patients were evaluated by using Dengue Non-structural Protein NS1 antigen test which is a rapid test kit. This has been used for the diagnosis in the remaining cases.

**Action Taken in the manuscript: **Necessary changes have been done in results section.

 4. **Comment: **What proportion of cases were dengue, severe dengue (DHF, DSS, Dengue with organ involvement - expanded Dengue syndrome)?

**Author’s Response:  **We concur that it is better to provide details regarding Dengue fever, DHF, DSS and Dengue with Organ involvement. As it is a hospital-based record-based study, we didn’t get all these details in the case record forms of the admitted patients. It is because of this reason we couldn’t analyse the data for determinants of dengue severity. Thus, this will be our study limitation.

 5. **Comment: **Conclusions: The conclusions are vague and tend to be written as general statements than from the results of the study.

**Author’s Response: **We are in complete agreement with your observation. Necessary changes have been done in the conclusion section of the manuscript.

**Action Taken in the manuscript: **Necessary changes have been done in conclusion section.
